# Social learning in large online audiences of health professionals: Improving dialogue with automated tools

**DOI:** 10.15694/mep.2019.000055.2

**Published:** 2019-06-26

**Authors:** Alvaro Margolis, Antonio López-Arredondo, Sofìa García, Nicolás Rubido, Camilo Caminada, Daniel González, Libertad Tansini

**Affiliations:** 1 Evimed; 2 Flacso; 3 School of Sciences; 4 School of Sciences; 5 School of Engineering; 6 School of Engineering

**Keywords:** Continuing Medical Education, MOOC, Social Network Analysis, Learning Analytics, Recommender Systems, Virtual Learning Environments, Social Networking applications, Facebook, Internet

## Abstract

This article was migrated. The article was marked as recommended.

Massive open online courses (MOOCs) bring about the opportunity to reach large international audiences of health professionals. However, change in clinical practice eventually needs social interaction, to validate the new knowledge with trusted peers, in the agreement and adoption phases of change. How can meaningful dialogue take place without scaling up expert tutoring?

The extensive experience from social network applications such as Facebook or Twitter provides an opportunity to improve dialogue among peers and with experts automatically and seamlessly, as part of what is called social learning analytics (SLA). Large amounts of data about prior relationships among participants in a course - similar to Facebook and other social applications-, among participants and course materials - similar to Netflix or Amazon -, as well as natural language processing, could be obtained, and then analyzed and used to improve the educational processes and outcomes.

In this paper, a series of examples with pilot uses of SLA in the context of massive online courses for physicians and other health care professionals are described. They include: 1) Forecasting of academic accomplishment. 2) Team-based face-to-face learning as part of massive online courses. 3) Analysis of existing connections, to ensure the most connected discussion groups of course participants. 4) Facebook-like dialogue with other course participants who are previously related, as well as with the Course Faculty. 5) *Crowdsourcing and friendsourcing*, for recommending useful study materials or future courses. 6) Natural language processing, to classify posts in online discussions. It should be noted that the article does not address the use of Facebook or Twitter in continuing medical education (CME), but instead, the use of their approaches in CME.

The intent of this manuscript is to create awareness in the medical education community that this type of analysis is possible and potentially useful, to receive feedback on the possible functionalities as well as critique these developments, and to create a space for collaboration in research and innovation projects with other interested parties.

## Introduction

Frequent chronic conditions pose a major burden to health care systems and societies across the world, in terms of morbidity, mortality and costs, both in developed and developing economies. For example, half of all U.S. adults have at least one chronic condition and 86% of health care spending is for patients with one or more chronic conditions ( Chapel *et al.,*2017). Moreover, burden of non-communicable diseases is greatest within low- and middle-income countries, where 78% of all of these deaths occur ( World Health Organization, 2018).

Massive open online courses (MOOCs) are open-access courses that allow for unlimited participation through the Web ( Kaplan and Haenlein, 2016). MOOCs bring about the opportunity to reach large professional audiences internationally, improving access to continuing education for health professionals worldwide. In fact, despite the challenge regarding its business model ( Walsh, 2014), the offering of MOOCs related to “Health and Medicine” in a course aggregator such as Class Central increased from 113 to 811 in the last 5 years ( Liyanagunawardena and Williams, 2014; Class Central, 2019). Still, most offerings are in English and are run by institutions from developed countries ( Liyanagunawardena and Williams, 2014).

Change in clinical practice eventually needs social interaction, to validate the new knowledge with trusted peers, in the agreement (i.e., when one accepts a new concept as valid) and adoption (i.e., when one starts applying it in clinical practice) phases of change (Davis, 2003). Furthermore, human interaction with peers as a main predictor for clinical impact is supported by the evidence, particularly active small-group discussions ( Mansouri and Lockyer, 2007).

Connectivist MOOCs encourage learning with and from peers ( Liyanagunawardena and Williams, 2014). Therefore, how could meaningful dialogue in a course take place, mimicking small group interaction, without scaling up expert tutoring?

Moreover, to add a level of complexity, frequent chronic conditions should be addressed by effective interprofessional practice, with collaboration and coordination by the different members of the health care team ( Goldberg and Crocombe, 2017; Coleman *et al*., 2009): so, how can interprofessional teams translate scientific knowledge regarding frequent chronic conditions to their local settings, from participation in large and international courses?

The extensive experience from social network applications such as Facebook or Twitter provides an opportunity to improve dialogue among peers and with experts automatically and seamlessly, as part of what is called social learning analytics (SLA). SLA focuses on how learners build knowledge together in their cultural and social settings (Ferguson and Buckingham- Shum, 2012). Conversations among learners are then paramount, with language as one of the primary tools through which learners construct meaning. As stated in (Ferguson and Buckingham- Shum, 2012), “understanding learning in these settings requires us to pay attention to group processes of knowledge construction”.

SLA includes several categories, such as *social network analytics,* with a focus on interpersonal relationships and their interaction in social platforms, *content analytics*, with user-generated and user-rated content as one of the defining characteristics of Web 2.0, or *discourse analytics*, with language as a primary tool for knowledge construction. Regarding the first category, social network analytics, large amounts of data about prior relationships among participants in a course - similar to Facebook and other social applications- could be applied to improve the educational processes and outcomes. Regarding the second category, content analytics, interactions between participants and course materials could be used, in a similar way to Netflix and Amazon. Regarding the last category, discourse analytics, natural language processing could be used with the same purpose.

In a previous publication ( Margolis and Parboosingh, 2015), one of the authors (AM) and a Canadian colleague analyzed the theoretical implications of the field of social learning analytics to continuing education in the health professions, as well as noted the scarce existing applications of it in Health Education.

In this paper, examples with pilot uses of SLA in the context of massive online courses for physicians and other healthcare professionals as well as teams are described. These examples are framed in the context of a series of massive online courses for Latin America ( Lombardi *et al*., 2018; Medina-Presentado *et al*., 2017; Margolis *et al*., 2015). It should be noted that the article does not address the use of Facebook or Twitter in continuing medical education (CME), but instead, the use of their approaches in CME.

The layout of the remainder of paper is as follows: First, ways to gather data about social network information from course participants will be shown, as well as how to process these data. Second, examples of uses of the network information collected and processed will be shown, together with the team necessary to implement these functionalities in online platforms. Afterwards, a discussion will follow.

## How to gather network data for social learning analytics

The first challenge of this new approach is to collect the data to be used for social learning analytics. In particular, the relationships among participants: when someone registers for an educational activity, usually demographic and professional information is collected, but no relationship data is sought regarding the rest of the participants in the activity (e.g., who do you work with, who do you know, who is your trusted peer, and so on). This information could be obtained manually or automatically: for large audiences, some kind of automated process needs to be in place.

In 2016, we first tested a semi-automated process in a course ( García *et al*., 2019; García, 2017): For the collection of data from the network (links between the participants) it was requested to course participants to authorize the system to access their Gmail contacts.

When the participant gave the authorization, the system connected with Gmail, matching the contacts of the person with the base of registered participants in the course.

The information that the system brought was then reflected in a list of participants (with name and photo) sorted by country, being the first country to appear that of the user who performed the process. The participant needed to confirm this list, being able to add or remove contacts if he or she wanted to. For this analysis we considered it as an undirected network, which means that if a participant marked another as a contact, the bidirectionality of the relationship was assumed.

The results from this strategy based on a non-mandatory request obtained 370 answers with contact information for 423 out of 601 participants in the selected course, and a total of 1148 links among them ( García *et al*., 2019; García, 2017). As we will see later, this result proved reasonably useful and was established as part of the operations for data collection for all of the subsequent courses.

A second major source of network information added later was group registration, mostly interprofessional teams from the same health care institution. It should be noted that a possible educational advantage of having teams register from the same institution is that, if face-to-face team study is promoted during the online course, scientific knowledge can be better applied in their specific setting; and to apply the chronic care model, interprofessional teams are needed ( Goldberg and Crocombe, 2017; Coleman *et al*., 2009). Therefore, collecting network data through group registration was in synergy with the overall educational design.

The type of connections obtained by group registration are qualitatively different too, because they are mostly from professionals working together on a daily basis: Trust is expected to be higher on average because of that reason ( Pentland, 2014), therefore social influence for changing behaviors after a course would be higher.

Group registration was promoted for courses from an educational and commercial standpoint, with lower registration rates for groups. Group registration in the first courses during 2017 was about half of all course registrations in most specialties, except in family and general medicine. See table 1.

**Table 1.** Group registration

Course	Main specialty	Total registrants	% group registration
Frequent infections in children	Pediatrics	679	62%
Management of the elderly patient in primary care	Family and general medicine	110	3%
Diabetes and Kidney	Nephrology	952	44%
Infection Control Management	Infectious diseases	898	53%

An additional source of information about connections has been group deliverables: educational activities that can be done by teams working in the same institution (not necessarily the same teams who registered together).

Later, recommendations for possible connections were added to the system, in a similar way as social network applications. A first way to do this, is to show the course participant other participants who know them, in order to offer them as possible contacts; additionally, contacts of your contacts are progressively offered in a similar way, as “people you may know”, similar to social network applications. Furthermore, if a participant sees interesting comments from another participant, he or she may add them as contacts, which is a way to make those comments more noticeable, similar to the “follow” function in some social network applications.

The information about connections persists with the participant in future courses: it does not need to be built from scratch for every course.

Finally, for content analytics, the interaction between participants and the educational platform needed to be obtained. Similarly, for discourse analytics, the exchange in the online discussions were necessary. Neither of them posed a problem, because they are readily available in any online platform.

## How to process the network information

A more complete description of the social network metrics that could be used for analysis is in ( Margolis and Parboosingh, 2015). In summary, at the individual level, each participant has a certain “ *degree centrality*”, referring to the number of connections the person has. Most social networks have opinion leaders (hubs, in network terms) with a disproportionate high number of connections. At the same time, the “ *betweenness centrality*” measures the number of times a person acts as a bridge between any two nodes: this kind of person is very important for disseminating knowledge across a network.

At the subnetwork level, the existence of “ *cliques*” is important: three or more people are connected by all possible connections (such as teams are: they all know each other). This is of practical interest for the improvement of the educational proposal, as this information allows the division of groups by affinity, rather than randomly.

At the network level, attributes such as “ *density*” can be measured: the proportion of existing connections relative to the total number of possible connections.

**Figure 1.**
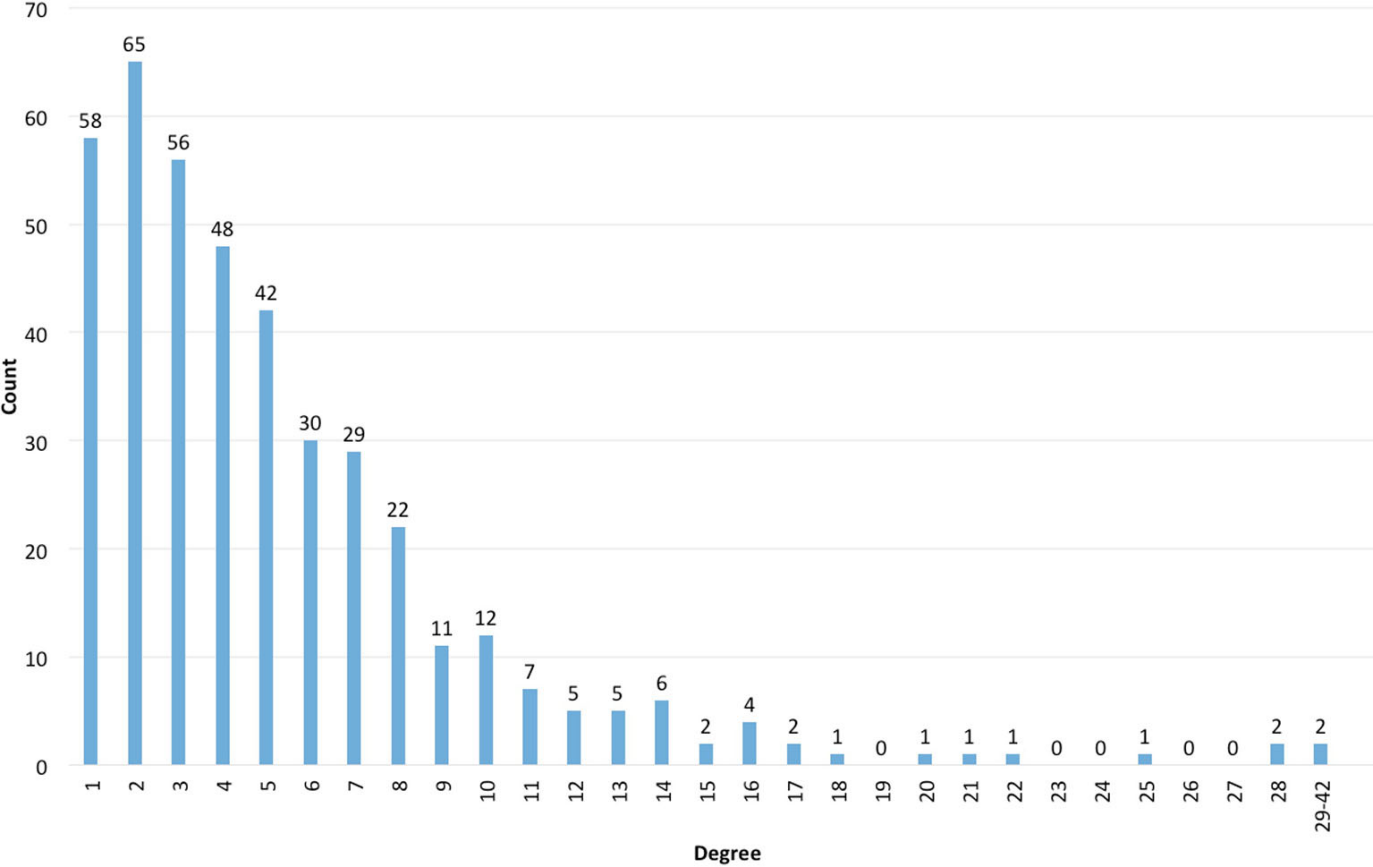
Degree histogram

As an example, regarding the degree distribution from the course mentioned before ( García *et al*., 2019; García, 2017), the information obtained is shown in table 2 and figure 1:

**Table 2.** Degree indicators

Indicator	n
Total nodes	423
Degree mean	6
Degree median	4
Degree mode	2
Minimum	1
Maximum	42

The distribution in this case is called “scale-free”, like the one Barabási ( Barabási, 2002) has described for social networks. In networks with such a distribution, most people have few contacts and few people have a very large number of contacts: they are named hubs, in network terms. This happens according to Barabási by the principle of preferential attachment, which indicates the tendency of people to connect with others that already have a greater number of connections. For example, in the Mathematics and Neuro-science research communities, the article collaboration networks evolve following preferential attachment - the new authors tend to search collaborations with the most referenced authors, which in turn continue to grow their already extensive co-authorship network ( Barabási, 2002b).

Furthermore, in the case of the network analyzed, 279 cliques were found, of the sizes shown in Table 3. They form the basis for group assignments, as mentioned before.

**Table 3.** Cliques in the network of a course (note: a node can be in more than one clique)

Amount of nodes in clique	Amount of cliques
3	119
4	80
5	52
6	21
7	7
Total Cliques	279

## The issue of data privacy and confidentiality

Before analyzing the possible uses of the information obtained, it should be noted that the protection of data privacy in the era of Internet and social network applications is of paramount importance: permission should be obtained from the users to collect the network data, the use should be restricted to the provision of a better learning experience and other approved uses by the recipients, it should not be used by third parties, and so on. And the national and regional regulations about data privacy and confidentiality in the territories where the educational activities are offered should be applied. On the other hand, these are closed health care education professional networks, which could offer a safer environment compared to widely available open global social network applications.

## What to do with the network information

Our initial interest was to use the network information as a potentially important (and hidden) variable in the forecast of academic achievement: i.e., less connected professionals would be more prone to being more passive and to drop out a course ( Margolis and Parboosingh, 2015).

The above-mentioned course was analyzed ( García *et al*., 2019; García, 2017), with the following results regarding prediction of access to educational contents, shown in table 4.

**Table 4.** Correlation coefficients between content access and network attributes

Correlation coefficient	Attribute
0,2068	Degree
0,1012	Neighborhood degree
0,0936	Betweenness coefficient
0,091	Eigenvector centrality
0,091	Hub or not
0,0642	Gender
0,0504	Country
0,036	Local clustering coefficient
0,0212	Workplace

Regarding content access, there was a statistically significant association between degree and access, although the correlation was weak. Similar results were obtained with the prediction of active participation through posts in discussions, and of course completion. Educational Data Mining was later applied with the use of these data, using decision trees that allowed to predict participation and course completion, using both network and available demographic data. The results showed that the accuracy of the algorithm was better using network data that without it. It implied that the fact of collecting network data was able to improve the prediction of the level of engagement of each participant, giving teaching tools for both human and automated tutoring.

A second and rather obvious use of network data was team-based face-to-face learning as part of these massive online courses. The fact of having many of the teams register as groups for the courses allowed to design work-based deliverables that could be addressed by these teams. For example, in a palliative course in nephrology, where physicians, nurses, psychologists, social workers and dieticians registered together, there was a two-week task where these teams had to develop a plan to improve palliative and supportive care for their own dialysis centers.

A third analysis, aimed at maximizing the likelihood of dialogue in online discussion groups, was to ensure that each of the online groups had participants with the highest number of connections, and that all groups were similar. This was done by running a Matlab program created for this purpose. This did not decrease the need to partition the audience in groups, and therefore did not alter the number of tutors needed for each of the courses, but maximized the existing connections in the groups that were created, with the potential for improved dialogue.

Specifically, this last analysis aims to group the participants into subgroups, each containing at least one participant with a high clustering coefficient and her/his closest contacts (namely, all the participants that share a connection with the highly clustered participant) while maximizing the overall density of connections for the resultant subgroups and the participants affinity. In this way, each subgroup is organized around (at least) one clique, maximizing the intra-group connections, and the resultant division of participants into the subgroups is chosen such that the average of connections is the maximum that can be achieved by other rearrangements of the participants and cliques.

A fourth functionality that was proposed, was to have a “Facebook-like” dialogue with other course participants who were previously related, as well as with the Course Faculty. In Facebook and other social network applications, there are no tutors, and each member sees the dialogue around the people they already know, as well as with the hubs or opinion leaders. To implement these functionalities generated a series of challenges, such as: how to display the discussion threads and weigh each variable (date of publication, Faculty or not, contact or not, number of likes, country of origin, and so on); how to show the discussion thread to the Faculty, and many other. But in the end, it allowed to have very large audiences moderated by only two tutors, as with smaller courses, and similar results in terms of participation, completion rates, learning, and satisfaction with the overall course and with the dialogue in the course. This “Facebook-like” dialogue eventually substituted the third strategy (group optimization), because groups were no longer used.

Regarding content analytics, recommendation algorithms which use different approaches such as *crowdsourcing and friendsourcing* were implemented during 2018 in order to recommend useful study materials, course activities and future courses ( González and Tansini, 2018).

The recommendations of resources, such as multimedia content, and activities such as discussion forums, was aimed to help course participants access a study material of potential interest. Also, the recommender system provided recommendations of comments published by other participants or Faculty, so they could be aware of the relevant discussions. These academic recommendations were aimed at enhancing the learning process in the educational platform. To generate the academic recommendations, the recommender system calculated for each user the relevance of the items, based on the evaluations of other colleagues who belonged to their network. All these recommendations were calculated in real time and offered to the participants during their interaction with the platform ( González and Tansini, 2018).

The recommendations of future courses, which were of interest to colleagues in their network, could also be of interest to the participants. To generate the “commercial” recommendations, the recommender system used demographic data to characterize the preferences of participants by specialty, gender, age or region, among others. All these recommendations were generated before the start of the courses and appeared once the users logged in to the platform as well as in the automatic emails that the learning platform sent to them ( González and Tansini, 2018).

Therefore, three algorithms were implemented: (1) recommendation of educational resources, (2) recommendation of comments in discussion forums, and (3) recommendation of future courses. Given the synchronic nature of algorithms 1 and 2 (the interaction with the user happens in real time in the educational platform) these offer the participant the possibility of giving feedback (Was the recommendation useful? YES or NO).

The statistics generated in the month of November 2018 for all ongoing courses show the following satisfaction rates of the users with the received recommendations, type 1 and 2:

• 98.3% for resources.
• 94.7% for comments.

Regarding the tailored recommendation of future courses using these algorithms (type 3), the automatic emails were compared with other type of massive email blasts:

1: generic emails that offer “many courses to many types of recipients”

2: course promotion messages signed and sent by recognized Faculty of the courses, addressed to a specific group of specialists.

3: highly personalized (with a recommendation of specific courses for each recipient, as a result of this innovation project).

An example of these three types of emails is shown in table 5, with the resulting statistics.

The higher conversion rate of the generic emails in comparison with the specific ones is probably due to the fact that many courses are offered in each email.

**Table 5.** Statistics of email recommendations, according to the type of email. See description in text.

Date	Type of email	Sent	Open	Clicks	Conversions (pre-registration or registration)	Conversion rate (conversion per 10,000 emails sent)
20/11/2018	Generic	27991	12,28%	2,08%	29	10,4
10/09/2018	Specific	21565	25,64%	1,89%	14	6,5
27/11/2018	Personalized by automation	12932	26,1%	1,64%	31	24,0

Regarding natural language processing, it was introduced to classify posts in online discussions. A commercial platform was used for this purpose ( MonkeyLearn, 2019). A new classifier system using this platform was trained to detect support requests in the clinical forums, and automatically derive them to support rather than post them in the clinical discussion. The participant was prompted of this possibility (to send a request to support or to otherwise post), and asked to confirm or not. Depending on the answer, the post was sent to the support system or published in the clinical forum.

Two other uses of natural language processing were tested, using the same platform: First, automatic language detection was already available and trained, and was introduced to classify posts in the clinical discussion and make sure the posts were in the language of the rest of the participants (namely, Spanish or Portuguese). This proved useful and continues in operation, along with the support/not support classifier system. Secondly, a classifier system for categorizing commitment-to-change statements ( Lockyer *et al*., 2001) was trained, but it did not classify these statements properly, which additionally would need to be course specific and are also difficult to categorize even for human beings, so it did not go into operations.

## Team necessary to develop the functionalities described above

The core team necessary to implement the functionalities described before consists of three profiles:

• The functional experts (physician educators and educational advisors) who understand the needs for Faculty and course attendees and define the functionalities that could be useful.
• The SLA experts (experts in the algorithms and artificial intelligence needed). This could vary from off-the-shelf machine learning programs where this knowledge is already embedded, such as the one we used for natural language processing, to tailor-made programs, such as the one we used for group optimization. The experts usually are software engineers, physicists or mathematicians, specialized in some of these areas (social network analysis, machine learning, natural language processing, among other).
• The IT professionals, who understand the needs from the functional experts and the implementation from the SLA experts, and add these functionalities in a learning platform.

In our experience, the functionalities are not well defined at the beginning so there is frequent trial and error process, and these three profiles have very different backgrounds and speak different professional languages. Therefore, the first challenge is to understand each other and grow a shared body of knowledge.

Other additional professional profiles could be included as well, such as a statistician for statistical analysis of the data obtained.

## Discussion

We are in a fast-changing world, shaped in the last decades by the advent of new information and communication technologies (ICT). As stated in ( Butler, 1997) “all modern forms of interaction-whether they be as simple as writing a letter or as complex as solving a problem in a team-are shaped by computing and communications technologies.” And interactions account for over one third of the economic activity in the United States and other developed regions, and a slightly smaller but also important part of developing economies. As forecasted in ( Butler, 1997), “convergence of technologies is set to increase our capacity to interact by a factor of between two and five in the near future. This enhanced interactive capacity will create new ways to configure businesses, organize companies, and serve customers, and have profound effects on the structure, strategy, and competitive dynamics of industries”. Online learning for CME is part of this wider technological revolution ( Walsh, 2019); for example, ICT allowed us to create an international network of academic institutions, experts and professionals, in order to deliver continent-wide continuing education, that would not have been possible two decades ago. It provides a digital help to the effort to bring together people with similar goals, and create and enhance networks overcoming space and time.

Interactions with peers and experts, in turn, are important for improving clinical practice through educational activities addressed to health care professionals. The ability to express one’s perceptions, emotions, and motivations, a process that leads to learning and behavior change ( Jordan *et al*, 2009), requires an environment that is perceived as relationally safe, one that is enclosed by trusted relationships with colleagues ( Wenger, 2009). When addressing large audiences, automated processes for this meaningful dialogue need to be considered.

Many of the functionalities described in this paper are based on a change of paradigm in the use of data, adding the relationships of the participants to the traditional demographic information. These new data then allow to create a series of features, such as the refinement of forecasting of academic accomplishment, workplace team learning, optimization of online groups, and creation of a personalized dialogue around each participant’s contacts and with the Faculty. Furthermore, having the focus on social learning supported by diverse SLA tools allows to recommend educational resources of potential interest to a certain participant, through crowdsourcing and friendsourcing, and to process natural language in order to improve conversations among participants in the courses.

Currently, many MOOCs use crowdsourcing methods to make posts with a higher rating more visible. However, health care professionals and teams have specificities in their needs compared to other industries, derived - for example - from the implementation of the chronic care model components related to professional and team education and coordination. Therefore, several other elements of SLA can be particularly useful, for example, those related to taking the participants’ networks into account, as shown in the examples before.

In a moment when several major MOOC platforms are aggressively entering into the CME field with paid courses and specializations ( Johnson, 2019), these specificities should be considered; experiences in the use of MOOCs for graduate medical education are also worth considering ( Marks and Meek, 2018).

As was mentioned before, an important clarification needs to be made: This article exemplifies how social network concepts and approaches can be used to promote online social learning in health care teams. However, it is not the use of Facebook or Twitter in CME, but instead, the use of their approaches in CME. The use of these existing social network applications is widespread, as they are practical, easy to use and free. However, there are some issues to be considered first: 1) the functionalities are predetermined by each platform (for example, Twitter is open, and Facebook groups have a certain way to operate, that cannot be controlled by the CME organizers). 2) They are for general use and open, so the issue of privacy and confidentiality is something to be considered. That is why we chose to use their concepts and approaches and build them into a learning platform instead.

Regarding the limitations of this paper, it should be noted that the examples are drawn from the experience with large online courses (i.e., usually between 500 and 1500 participants), that are open to participation by the target audiences covered by each course but *not free* ( Lombardi *et al*., 2018; Medina-Presentado *et al*., 2017; Margolis *et al*., 2015). The fact of having a registration fee impacts on the participation and completion rates, which are much higher compared to traditional MOOCs, but the challenge for meaningful dialogue is still present, as in any very large online course. And, as noted before, the number of paid courses for health professionals in MOOC platforms will probably increase dramatically, as Coursera, Edx and other platforms are currently getting heavily involved in paid CME ( Johnson, 2019).

Another limitation is that these courses covered basically *Latin America*, a region with 600 million people and one million physicians, as well as other health care professionals, who speak mainly Spanish and Portuguese and live in over 20 diverse middle-income countries; as such, it may be considered a testbed for similar international programs for health professionals in other regions.

## Conclusions

The intent of this manuscript is to create awareness in the medical education community that social learning analytics in CME is possible and potentially useful, to receive feedback on the possible functionalities as well as critique these developments, and to create a space for collaboration in research and innovation projects with other interested parties.

## Sample algorithms

Trial of some of the algorithms described in the paper, at:

https://redemc.net/algorithms/

Sample massive course (in Spanish), where these algorithms were implemented:

https://atbgrama2018.evimed.net

User: MedEdPublish

Password: February2019

## Take Home Messages

• Social learning is important to produce changes in clinical practice.
• Social learning in large groups can be improved with automated tools, already used by social networking applications such as Facebook and others, but still underdeveloped in online continuing education for the health professionals.
• There is a need for collaboration among public and private institutions internationally to develop this area of research.

## Notes On Contributors

Alvaro Margolis is an internist with a Master’s degree in Medical Informatics from the University of Utah (USA). He has held academic positions at the Schools of Medicine and Engineering, Universidad de la República, Uruguay, is Founding Member of the International Academy of Health Sciences Informatics, and Associate Editor of Applied Clinical Informatics. He is the President of the Global Alliance for Medical Education (GAME), and Director of EviMed, a CME company working across Latin America. ORCID: https://orcid.org/0000-0002-2631-2323

Antonio López-Arredondo is Adjunct Professor at the Health Informatics Laboratory, Universidad de la República, Uruguay. He is also managing partner at EviMed.

Sofía García holds a Master’s degree in educational processes & technologies. She is a teacher and advisor in e-learning at FLACSO Uruguay, educator across Latin America for the AO Foundation and researcher in Ceibal Foundation (OLPC Uruguay). She has extensive experience in on-line CME courses in Latin America.

Nicolás Rubido is Adjunct Professor of the Physics Institute in the School of Sciences (IFFC) at the Universidad de la República, Uruguay. He is also Level 3 researcher at PEDECIBA (Program for the Development of Basic Sciences) and Level 1 researcher at the National System of Researchers (SNI, ANII). ORCID: https://orcid.org/0000-0002-0616-2479

Camilo Caminada is a software programmer at EviMed.

Daniel González is a Computer Engineer and a master’s student at the Program for the Development of Basic Sciences (PEDECIBA), Universidad de la República, Uruguay. His main areas of research are Recommender Systems and Social Collaborations in Virtual Learning Environments.

Libertad Tansini is Associate Professor at the Computer Science Department, Universidad de la República, Uruguay. She pursued her PhD studies in Chalmers University of Technology, Sweden, and has since then done research in the field of data mining to improve user experience in web search and recommendations.
