# Supplementary material for: Social learning in large online audiences of health professionals: Improving dialogue with automated tools
Source: MedEdPublish (2016). 2019 Jun 26;8:55. Originally published 2019 Mar 14. [Version 2] doi: 10.15694/mep.2019.000055.2 (PMC10712608; doi:10.15694/mep.2019.000055.2)
Supplement: Peer review history

# Reviewer report: Reviewer response for version 2

**DOI:** 10.21956/mep.20243.r31389

**Published:** 2019-10-22

**Authors:** Ken Masters

**Affiliations:** 1 Sultan Qaboos University

This review has been migrated. The reviewer awarded 5 stars out of 5 This is Version 2 of an already well-written paper on social learning in large online audiences of health professionals, with an emphasis on improving dialogue with automated tools. This is a really interesting and useful paper for those who wish to explore analytics beyond simple number of downloads and accesses. Personally, I would like to have seen a little more discussion on the way in which the analysis extends and reinforces the original vision of the constructivism of the original MOOCs, especially in medical education; as this aspect was not, however, requested by the reviewers of Version 1, I do not hold it as a weakness of this Version – perhaps the authors may be encouraged to write another paper on that topic.Overall, sound groundwork on which others may build.

Reviewer Expertise:

NA

No decision status is available

# Reviewer report: Reviewer response for version 2

**DOI:** 10.21956/mep.20243.r31390

**Published:** 2019-07-03

**Authors:** John Parboosingh

**Affiliations:** 1 University of Calgary, Canada

This review has been migrated. The reviewer awarded 5 stars out of 5 Responding to suggestions made by reviewers of the first version of this (already excellent) paper, the authors have created a well written report on original research of the highest quality on a topic of great importance to the development of continuing medical education (CME). The authors rightly recognize their work has the potential to spearhead transformative change in accredited CME. The research builds on technologies used for years by companies that recognized the role consumer interactivity plays in fostering behavior change; and observations that grouping learners with similar practices builds trusting relationships and creates a safe environment that fosters peer-to-peer as well as peer-to-expert interactivity from which deep learning and changes in behavior may result. The paper describes how technologies can be used to enhance the effectiveness of massive open online courses (MOOCs) which provides a ‘research laboratory’ to change traditional CME in the direction reports from evaluative studies recommend to improve practitioner performance and patient outcomes. For instance, designers of MOOCs, cognizant of the role of social learning in behavior change recruited experts to design online platforms that encourage interactivity but have proved limited in building trusting relationships and safe environments for learners. This report describes how a team of experts from different fields explored and programmed social learning analytic tools to recognize learners with similar interests and practices and created groups of learners who have already built trusted relationships from prior contacts. The lead author is to be congratulated for creating and leading a team of professionals from different backgrounds to produce this unique research initiative. This paper encourages readers to vision future directions for research and development in CME, licensure and Maintenance of Certification (MOC). For instance, we can use learning analytics to determine active ingredients and set quality standards for conversations that lead to changes in behavior. Working with experts in Artificial Intelligence and Information Technologies, as these researchers have, we can explore the use of new educational technologies, such as Experience API, to track learning activities and digital badges, to recognize achievements of learners, advances that could make significant contributions to competency-based CME and MOC.

Reviewer Expertise:

NA

No decision status is available

# Reviewer report: Reviewer response for version 1

**DOI:** 10.21956/mep.19122.r27601

**Published:** 2019-03-27

**Authors:** Helena Filipe

**Affiliations:** 1 Hospital of the Armed Forces/PL-EMGFA

This review has been migrated. The reviewer awarded 5 stars out of 5 Despite the increasing use and acceptance within academia, of social media in medical education, robust evidence of its clear advantage is scarce. Research is then more than welcome and thus Alvaro and Associates article is highly welcome.Using Facebook and Twitter in medical education is widespread as practical, widely used, user friendly and free. As Web 2 tools, their use promote collaboration and the sense of learning community for those united with similar goals. Social learning, constructivism, connectivism and self-determined learning are educational theories closely related and a reliable foundation for the application of social media in continuing medical education.Social learning analytics (SLA) is an interesting methodology as it represents a digital help to the analogic effort to bringi together people with similar goals and create and enhance fruitful networks overcoming space and time. This methodology becomes highlighted within the MOOCs by bringing together learners with more specific learning needs within the courses’ goals, mimetizing the “human factor” and facilitating the creation of learning communities.By sharing their experience, and translating medical theory into digital educational methodology the Authors are also already embracing the new challenges of the future CPD educator farther from the “sage on the stage” and much closer to the sage facilitator.The Authors start by a robust background to follow with the methods they used for collecting and processing data and creating social learning groups. They make an approach to substantiate statically their purpose and conclusion. The team needed to carry on this type of work is inter-professional and collaborative.A synthesis of results and a longer elaboration in the discussion section would be acceptable; such is the complexity of the topic.Within their line of thought and vision the Authors also bring an exciting invitation to the interested reader to join with experience and future projects in this field.I hope the Authors progress their already vast experience with future work and research and that their next paper will show us a step ahead of awareness of these analytics methodology.

Reviewer Expertise:

NA

No decision status is available

# Reviewer report: Reviewer response for version 1

**DOI:** 10.21956/mep.19122.r27602

**Published:** 2019-03-26

**Authors:** Jann Balmer

**Affiliations:** 1 University of Virginia

This review has been migrated. The reviewer awarded 5 stars out of 5 This article has successfully discussed one of the major challenges for continuing education in the health professions: the application of learning and new information into clinical practice. The ability of learners in a MOOC environment to build communities of practice and to build interprofessional collaboration that can be integrated into practice is an important advancement in the field. The approach is unique and can create a new framework for the design and broad dissemination of standards of care that can positively affect populations regardless of their geographic location.

Reviewer Expertise:

NA

No decision status is available

# Reviewer report: Reviewer response for version 1

**DOI:** 10.21956/mep.19122.r27600

**Published:** 2019-03-17

**Authors:** John Parboosingh

**Affiliations:** 1 University of Calgary, Canada

This review has been migrated. The reviewer awarded 5 stars out of 5 John ParboosinghThe authors describe techniques to foster interactivity in massive open online courses (MOOCs) with potential to enhance participant engagement and change in practice; and, as proof of concept, they report the results from several pilot courses. MOOCs provide educators with a ‘research laboratory’ to change traditional continuing medical education (CME) in the direction reports from evaluative studies recommend to improve practitioner performance and patient outcomes (Cervera RM and Gaines JK, 2015). For instance, MOOCs allow participation at the practitioners’ convenience and enable on-going interactivity between learners as occurs in communities of practice (Wenger E. 1998). Recognizing the social component involved in changing practice, the authors explore innovative ways of grouping learners to enhance interactivity. I suggest the authors emphasize this point, for instance by inserting the sentence, “the ability to freely express one’s perceptions, emotions, and motivations while recalling a practice experience, a process that leads to learning and behavior change, (Jordan ME et al 2009) requires an environment that is perceived as relationally “safe,” one that is enclosed by trusted relationships with colleagues. (Wenger E. 2009).The authors rightly recognize their work has the potential to spearhead transformative change in accredited CME. They build on technologies developed for social media and companies that decades ago recognized the role consumer interactivity plays in fostering behavior change (Butler P. et al 1997). The lead author is to be congratulated for creating and leading a team of professionals from different backgrounds to produce this unique research initiative. This paper provides us with a glimpse into the future for CME and Maintenance of Certification (MOC). For instance, we can use learning analytics to determine active ingredients and set standards for conversation that leads to changes in behaviors. Working with experts in Artificial Intelligence and Information Technologies, we can explore the use of new educational technologies, such as Experience API to track learning activities and digital badges to recognize achievements of learners, advances that could make significant contributions to competency-based CME and MOC.ReferencesCervero RM, Gaines JK. Journal of Continuing Education In The Health Professions, 35(2):131–138, 2015.Wenger E. Communities of Practice: Learning, Meaning, and Identity. Cambridge, UK; New York, NY: Cambridge University Press; 1998.Jordan ME, Lanham HJ, Crabtree BF, et al. The role of conversation in health care interventions: enabling sensemaking and learning. Implement Sci. 2009:4:1–15.Wenger E. Four essays on innovation and learning in social systems: social learning capability. 2009. http://wenger-trayner.com/wp-content/uploads/2011/12/09-04-17-Social-learning-capability-v2.1.pdfButler P. et al. A Revolution in Interaction. The McKinsey Quarterly 1997 (1).

Reviewer Expertise:

NA

No decision status is available
